# NaGd(MoO_4_)_2_ nanocrystals with diverse morphologies: controlled synthesis, growth mechanism, photoluminescence and thermometric properties

**DOI:** 10.1038/srep31366

**Published:** 2016-08-10

**Authors:** Anming Li, Dekang Xu, Hao Lin, Shenghong Yang, Yuanzhi Shao, Yueli Zhang

**Affiliations:** 1 State Key Laboratory of Optoelectronic Materials and Technologies, School of Materials Science and Engineering/School of Physics, Sun Yat-sen University, Guangzhou, 510275, China; 2 Institute of Optoelectronic Engineering, Department of Optoelectronic Engineering, Jinan University, Guangzhou, 510632, China

## Abstract

Pure tetragonal phase, uniform and well-crystallized sodium gadolinium molybdate (NaGd(MoO_4_)_2_) nanocrystals with diverse morphologies, e.g. nanocylinders, nanocubes and square nanoplates have been selectively synthesized *via* oleic acid-mediated hydrothermal method. The phase, structure, morphology and composition of the as-synthesized products are studied. Contents of both sodium molybdate and oleic acid of the precursor solutions are found to affect the morphologies of the products significantly, and oleic acid plays a key role in the morphology-controlled synthesis of NaGd(MoO_4_)_2_ nanocrystals with diverse morphologies. Growth mechanism of NaGd(MoO_4_)_2_ nanocrystals is proposed based on time-dependent morphology evolution and X-ray diffraction analysis. Morphology-dependent down-shifting photoluminescence properties of NaGd(MoO_4_)_2_: Eu^3+^ nanocrystals, and upconversion photoluminescence properties of NaGd(MoO_4_)_2_: Yb^3+^/Er^3+^ and Yb^3+^/Tm^3+^ nanoplates are investigated in detail. Charge transfer band in the down-shifting excitation spectra shows a slight blue-shift, and the luminescence intensities and lifetimes of Eu^3+^ are decreased gradually with the morphology of the nanocrystals varying from nanocubes to thin square nanoplates. Upconversion energy transfer mechanisms of NaGd(MoO_4_)_2_: Yb^3+^/Er^3+^, Yb^3+^/Tm^3+^ nanoplates are proposed based on the energy level scheme and power dependence of upconversion emissions. Thermometric properties of NaGd(MoO_4_)_2_: Yb^3+^/Er^3+^ nanoplates are investigated, and the maximum sensitivity is determined to be 0.01333 K^−1^ at 285 K.

Nowadays, lanthanide-doped nanocrystals, especially upconversion nanocrystals, have become the current focus of intensive researches due to their unique photoluminescence properties and consequently numerous applications, such as bio-imaging and bio-probe, photodynamic/chemo-therapy, drug delivery, temperature sensing, solar cells, optoelectronics and photocatalysis^1^,^2^,^3^,^4^,^5^,^6^,^7^,^8^,^9^,^10^. Compared with the luminescence from conventional organic dyes or quantum dots, lanthanide luminescence from nanocrystals exhibits many advantages, including high photostability (high resistance to optical blinking and photobleaching), large Stokes/anti-Stokes shifts, sharp emission bandwidths, abundant emission channels, long excited-state lifetime, low cytotoxicity, and low synthesis expenditure^11^,^12^,^13^,^14^,^15^. All these merits endow lanthanide-doped nanocrystals with efficient luminescence, high detection sensitivity and signal-to-noise ratio, and ease of use in aforementioned applications. The morphology (size and shape) of nanocrystals will affect their physicochemical properties, and the synthesis of nanocrystals with tunable morphologies is particularly significant for the applications in biological and biomedical fields^16^,^17^,^18^. So morphology-controlled synthesis of nanocrystals has attracted much attention from researchers.

Double alkaline rare-earth molybdates ARe(MoO_4_)_2_ (A = alkali metal cation, Re = trivalent rare-earth metal cation) have been demonstrated to be promising candidates as luminescent host materials for numerous applications, due to their favorable chemical and physical stability, large lanthanide admittance, and relatively low phonon energy^19^,^20^,^21^,^22^,^23^. Many researches have been devoted to the synthesis and luminescence properties of lanthanide-doped molybdate microcrystals or phosphors^24^,^25^,^26^,^27^,^28^,^29^. Nevertheless, the synthesis or luminescence properties of double alkaline rare-earth molybdate nanocrystals are rarely reported, which result from the faster crystallization and growth rate and difficulty in controlling the growth process of double molybdates^30^,^31^. Bipyramid-like NaLa(MoO_4_)_2_: Eu^3+^ nanocrystals were synthesized hydrothermally using oleic acid/oleylamine as surfactant^32^. NaLa(MoO_4_)_2_: Eu^3+^, Eu^3+^/Tb^3+^ shuttle-like nanorods composed of nanoparticles were prepared hydrothermally using ethylene glycol as ligand and their luminescent properties were discussed^33^,^34^. However, most of these works focused on either nanocrystals with only a single morphology or poor-crystallized composite nanoparticles, and controlled synthesis of double molybdates nanocrystals with diverse morphologies has not been reported so far.

Solution-based wet chemical methods, which allow a fine control of size, shape and chemical homogeneity of the products by fine tuning of experimental conditions, are universally employed to synthesize nanocrystals^35^,^36^. Some organic additives with functional groups or long hydrocarbon chains (e.g. oleic acid, citrate acid, oleylamine, ethylenediamine tetraacetic acid, and cetyltrimethyl ammonium bromide) can act as complexing agents and shape modifier by adjusting the growth rate of different facets under hydrothermal conditions^37^,^38^. In this work, we present a novel template-free morphology-controlled hydrothermal synthesis of NaGd(MoO_4_)_2_ nanocrystals. Pure tetragonal phase, uniform and well-crystallized NaGd(MoO_4_)_2_ nanocrystals with several distinct morphologies, including nanocubes and square nanoplates, can be selectively synthesized by a modified hydrothermal method using oleic acid as complexing agent. The morphology of the synthesized NaGd(MoO_4_)_2_ nanocrystals can be controlled by simply tuning the contents of oleic acid in the precursor solution. Effects of oleic acid and sodium molybdate (Na_2_MoO_4_) on the formation of NaGd(MoO_4_)_2_ nanocrystals and growth mechanism of NaGd(MoO_4_)_2_ nanoplates are discussed. Meanwhile, morphology-dependent down-shifting photoluminescence properties of NaGd(MoO_4_)_2_: Eu^3+^ nanocrystals, upconversion photoluminescence properties of Yb^3+^/Er^3+^ and Yb^3+^/Tm^3+^ square nanoplates, and thermometric properties of NaGd(MoO_4_)_2_: Yb^3+^/Er^3+^ square nanoplates are investigated in detail.

## Results and Discussion

### Crystal structures, morphologies and compositions

**Figure 1:**
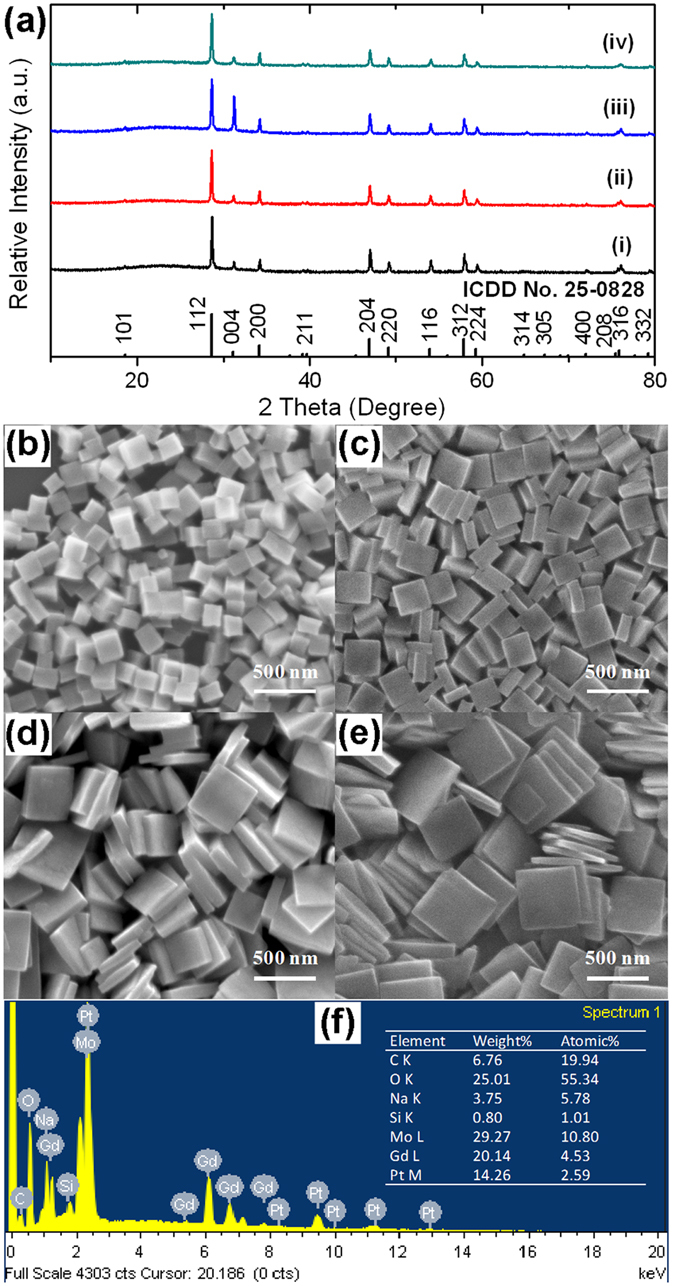
(**a**) XRD patterns and (**b–e**) SEM images of the NaGd(MoO_4_)_2_ nanocrystals with four typical morphologies; (**f**) EDS spectrum of the NaGd(MoO_4_)_2_ square nanoplates.

NaGd(MoO_4_)_2_ nanocrystals with specific uniform morphologies are synthesized hydrothermally at 180 °C for 12 h with different contents of oleic acid (0.25, 0.75, 1.25 and 1.5 ml) and 10 mmol Na_2_MoO_4_ in the initial precursor solutions. Figure 1a shows the XRD patterns of NaGd(MoO_4_)_2_ nanocrystals samples with four typical morphologies, including nanocubes (pattern i) and square nanoplates with different aspect ratios (patterns ii–iv), in the 2 theta range of 10–80°. All the diffraction peaks can be readily indexed to a tetragonal phase NaGd(MoO_4_)_2_ (ICDD No. 25-0828). The absence of any other phases in the XRD patterns indicates high purity of the as-synthesized NaGd(MoO_4_)_2_ nanocrystals. The strong and sharp diffraction peaks indicate high crystallinity. The cell parameters for the four patterns calculated by using a refinement program of Jade 5.0 software are: *a* = *b* = 5.24476 Å, *c* = 11.44005 Å, *V* = 314.69 Å^3^ for pattern i; *a* = *b* = 5.24906 Å, *c* = 11.45052 Å, *V* = 315.49 Å^3^ for pattern ii; *a* = *b* = 5.24126 Å, *c* = 11.43870 Å, *V* = 314.23 Å^3^ for pattern iii; and *a* = *b* = 5.24649 Å, *c* = 11.45342 Å, *V* = 315.26 Å^3^ for pattern iv, which agree well with literature values of NaGd(MoO_4_)_2_ (ICDD No. 25-0828). The average crystallite sizes calculated using Scherrer’s formula from the broadening of the diffraction peak (112) in the four patterns are 58, 51, 48 and 49 nm, respectively. What’s more, compared with the standard diffraction data, quite intense (004) diffraction peak is found in the pattern iii of Fig. 1a, indicating a preferentially oriented crystallization might exist along the (001) planes of NaGd(MoO_4_)_2_ nanocrystal. Due to the morphological characters of the samples and discrepancy in sample preparation procedure for the XRD measurements, the enhancement of (004) peaks in patterns ii and iv is not evident.

The SEM images of the NaGd(MoO_4_)_2_ nanocrystals with four typical morphologies were shown in Fig. 1b–e. Obviously, all the samples exhibit uniform, regular and well-crystallized nanocrystals. In Fig. 1b, the sample consists of monodisperse and uniform nanocubes with side length of ~150 nm. In Fig. 1c–e, the samples are composed of monodisperse and uniform square nanoplates. The thicknesses are about 85, 70, 50 nm and the side lengths are about 250, 400 and 500 nm for the samples in Fig. 1c–e, respectively. The chemical composition of the NaGd(MoO_4_)_2_ nanocrystals with the morphology of square nanoplates (shown in Fig. 1d) was analyzed by the EDS spectrum shown in Fig. 1f. The sample is confirmed to be composed of Na, Gd, Mo and O. The measured atomic ratio of Na, Gd, Mo and O is close to the stoichiometric proportion of NaGd(MoO_4_)_2_. C peak and excessive proportion of O come from a little oleic acid adsorbed on the surface of the sample. The Si and Pt peaks arise from silicon substrate and conductive coating.

**Figure 2:**
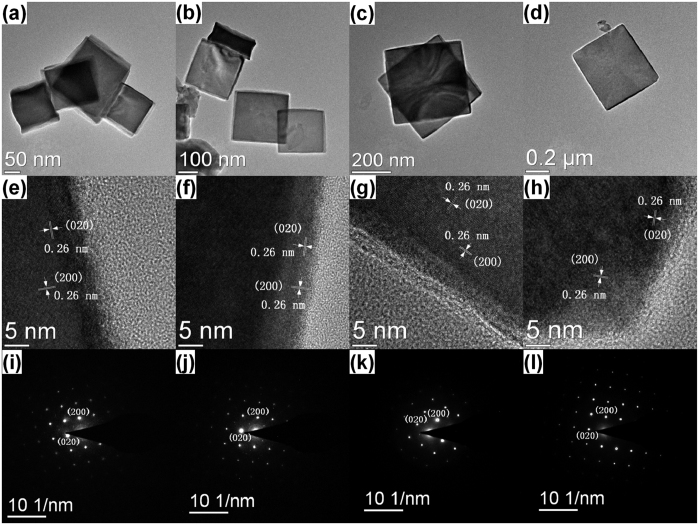
(**a–d**) TEM images of the NaGd(MoO_4_)_2_ nanocrystals with four typical morphologies; (**e–h**) HRTEM images and (**i–l**) SAED patterns of the NaGd(MoO_4_)_2_ nanocrystals with four typical morphologies taken in [001] incidence of a nanocube/nanoplate.

More details about the morphological and structural features were further investigated by employing TEM, HRTEM and SAED. The morphologies of the samples shown in the TEM images (Fig. 2a–d) are consistent with that in SEM images. The HRTEM images taken at the edge of the nanocube/nanoplates (Fig. 2e–h) reveal perfect crystalline surfaces. The interplanar distances between adjacent lattice fringes of the four samples are all about 0.26 nm, which correspond to the *d* spacing of (200) or (020) planes of tetragonal NaGd(MoO_4_)_2_ structure. These lattice fringes indicate that the nanoplates grow along [100] and [010] directions, namely (001) planes, which agrees well with the speculation from the XRD analysis. Taking the morphology of the nanocrystals (square plate) into account, it is also inferred that the normal direction of the upper surface of the square nanoplates is the zone axis ([001] orientation). Thus the upper surface of the square nanoplates belongs to (001) planes of tetragonal NaGd(MoO_4_)_2._ The SAED patterns of all the samples show highly ordered sharp spots, which indicate the single crystalline nature of the samples. The diffraction dots are indexed to (200) and (020) planes of tetragonal NaGd(MoO_4_)_2_.

FTIR analysis was performed to investigate the surface properties of the samples. Supplementary Fig. S1 presents the FTIR spectra of the NaGd(MoO_4_)_2_ nanocrystals with four typical morphologies (Fig. 1b–e). The spectra are similar in shape. A broad band at about 3399 cm^−1^ corresponds to the O–H stretching vibrations is observed, arising from surface-adsorbed ambient water. Small peaks at about 2927 and 2856 cm^−1^ are attributed to the stretching vibration of –CH_2_ in skeletal chain of oleic acid. The peaks at about 1635 and 1460 cm^−1^ are ascribed to the vibrations of the C = O groups of oleic acid^39^. The strong absorption bands at 796 and 704 cm^−1^ are assigned to the F_2_ (ν_3_) antisymmetric stretch and the peak at 434 cm^−1^ is ascribed to F_2_ (ν_4_) bending mode vibrations related to the O–Mo–O stretching vibrations in the MoO_4_ tetrahedron^40^. These results show the existence of residual complexing ligand on the surface of the samples.

### Formation of the NaGd(MoO_4_)_2_ nanocrystals

#### Effects of Na_2_MoO_4_ on the morphology of the NaGd(MoO_4_)_2_ nanocrystals

Generally speaking, the molar ratio of the starting source reagents in the precursor solutions would affect the morphology and/or phase of the products in the hydrothermal synthesis procedure. To investigate the effects of the Na_2_MoO_4_ on the morphology of the synthesized NaGd(MoO_4_)_2_ nanocrystals, NaGd(MoO_4_)_2_ samples are synthesized with different amounts of Na_2_MoO_4_ varying from 2 to 12 mmol, and the fixed amount of oleic acid (1.25 ml) in the precursor solution. Supplementary Figs S2 and S3 present the XRD patterns and SEM images of the as-synthesized samples. From the XRD patterns, it is observed that all the samples are pure tetragonal phase NaGd(MoO_4_)_2_ (ICDD No. 25-0828). Similarly, intense diffraction peak (004) is also observed in pattern c of Supplementary Fig. S2. As can be seen from the SEM images, the samples are comprised of nanoparticles and bipyramids when a small amount of Na_2_MoO_4_ is added. With the increasing amount of Na_2_MoO_4_, the samples evolve toward square nanoplates. And both the side length and thickness of the square nanoplates are reduced with the increase of Na_2_MoO_4_ content (Supplementary Fig. S3c–e). The sample exhibits irregular nanoflakes with nearly round shape at further increasing Na_2_MoO_4_ content (12 mmol, Supplementary Fig. S3f).

According to Bravais-Friedel-Donnay-Harker theory, high-index facets with high surface free energy have a large growth rate and will not be expressed in the equilibrium morphology of the resulting crystal^41^. Based on crystal structure models of tetragonal NaGd(MoO_4_)_2_ shown in Supplementary Fig. S4, packing density of Gd^3+^/Na^+^ for some low-index facets are calculated to be 0.0364 Å^−2^ for {001} facets, 0.0332 Å^−2^ for {010}/{100} facets and 0.0227 Å^−2^ for {101} facets. The packing densities of Gd^3+^/Na^+^ on the {001} facets are higher than that on other facets. Na_2_MoO_4_ will ionize and provide (MoO_4_)^2−^ anions in the hydrothermal solution. When excess (MoO_4_)^2−^ exist in the solution, it will be preferentially adsorbed on {001} facets of tetragonal NaGd(MoO_4_)_2_ nanocrystal nuclei due to the strong electrostatic interaction between (MoO_4_)^2−^ and Gd^3+^/Na^+^ 42. This preferential adsorption of (MoO_4_)^2−^ on {001} facets will reduce the growth rate along [001] directions and cause preferentially oriented crystallization along the (001) plane in the NaGd(MoO_4_)_2_ nanocrystals. So the samples exhibit thinner square nanoplates at higher Na_2_MoO_4_ contents. A slightly lower adsorption energy of (MoO_4_)^2−^ for the other facets than that for {001} facets will also cause the adsorption of (MoO_4_)^2−^ on the other non-{001} facets, but the restriction of the growth rate is weaker than that for {001} facets. Thus the side length is slightly reduced with increasing Na_2_MoO_4_ content. More excess amounts of Na_2_MoO_4_ will provide more (MoO_4_)^2−^ anions, and break the equilibrium of growth dynamics for the nanocrystals and make the products more irregular.

#### Effects of oleic acid on the formation of NaGd(MoO_4_)_2_ nanocrystal

**Figure 3:**
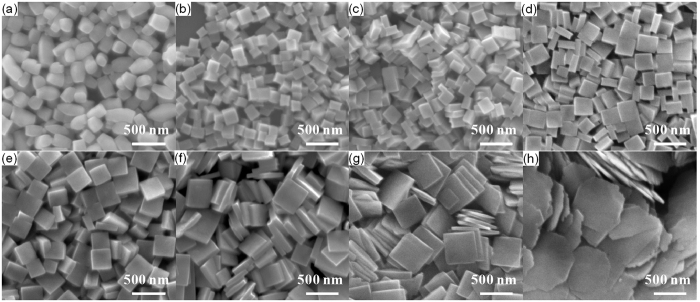
SEM images of the NaGd(MoO_4_)_2_ nanocrystals synthesized with different contents of oleic acid: (**a**) 0 ml, (**b**) 0.25 ml, (**c**) 0.5 ml, (**d**) 0.75 ml, (**e**) 1 ml, (**f**) 1.25 ml, (**g**) 1.5 ml, (**h**) 1.75 ml.

As is previously discussed, appropriate amount of Na_2_MoO_4_ favor the formation of the synthesized NaGd(MoO_4_)_2_ nanocrystals with regular morphology. So the amount of Na_2_MoO_4_ is further fixed at 10 mmol and NaGd(MoO_4_)_2_ nanocrystal samples synthesized with different contents of oleic acid (0–1.75 ml) in the initial precursor solution. As can be seen from the XRD patterns shown in Supplementary Fig. S5, all the diffraction peaks of the as-synthesized samples can be easily indexed as pure tetragonal phase NaGd(MoO_4_)_2_ (ICDD No. 25-0828). No peak from impurities or other phases can be found in these patterns, indicating high purity of the samples. A relatively intense diffraction peak (004) is observed in pattern f (Supplementary Fig. S5). Figure 3 shows the SEM images of the synthesized NaGd(MoO_4_)_2_ nanocrystal samples. When no oleic acid is added, the products exhibit barrel-like nanocylinders with the height of ~300 nm and diameter of ~200 nm (Fig. 3a). Whereas when oleic acid was added into the reaction system, new morphologies appear. Nanocubes were obtained when 0.25 ml oleic acid was added (Fig. 3b). When the content of oleic acid is increased to 0.5 ml, the morphology of the products varies from nanocubes to square nanoplates (Fig. 3c). With the increase of oleic acid content ranging from 0.5 to 1.5 ml, the morphologies of the products are all square nanoplates, whereas the thickness of the nanoplates decreases and the side length increases gradually (Fig. 3c–g). The square nanoplates become increasingly thinner and flatter with the increase of oleic acid contents, and seem to be squashed gradually. When 1.75 ml oleic acid is added, the products become irregular thinner nanoflakes with near-circular shape and thickness less than 50 nm (Fig. 3h).

It is noted that oleic acid acts as complexing agent and shape modifier, and plays a key role in the morphology-controlled synthesis of NaGd(MoO_4_)_2_ nanocrystals. When lanthanide nitrates solution is added into the mixed solution of oleic acid and ethanol, lanthanide oleate complexes ((RCOO)_3_Ln) are formed through strong coordination interaction and ion exchange process. The oleate complexes could control the concentration of free Ln^3+^ in solution and thus help to control the growth of the nanocrystals in a dynamical view^43^. In addition, the oleic acid in the hydrothermal solution will limit the growth rate of specific planes of the nanocrystals through interactions with lanthanide ions^44^. In our case, oleate ions are supposed to be selectively adsorbed on the {001} facets of square NaGd(MoO_4_)_2_ nanocrystals. The adsorbed oleate ions will reduce the reactivity of the {001} facets and limit the growth rate along the [001] direction (perpendicular to the {001} planes) of NaGd(MoO_4_)_2_ nanocrystals. Therefore, the more amount oleic acid is added, the more (001) and 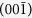 facets are expressed in the eventual equilibrium morphologies of nanocrystals, which will result in the formation of NaGd(MoO_4_)_2_ square nanoplates. When excess amounts of oleic acid is added (1.75 ml or more in our case), the growth kinetics will be changed. A more reduced crystal growth rate along the [001] directions means a relatively faster growth rate along (001) planes (i.e. along [100] and [010] directions). A faster and faster growth rate along (001) planes will make the growth behavior out of kinetic control and lead to the formation of irregular near-circular nanoflakes. Therefore, the morphology of the as-synthesized NaGd(MoO_4_)_2_ nanocrystals evolves in the sequence of nanocylinders, nanocubes, square nanoplates and irregular nanoflakes with the increase in the oleic acid content.

#### Growth mechanism of NaGd(MoO_4_)_2_ nanocrystals

It is hard to observe the crystallization process in the hydrothermal apparatus directly and the growth mechanism of hydrothermally synthesized nanocrystals is generally inferred from the morphology observation and XRD analysis of the products obtained at different reaction time intervals. Taking NaGd(MoO_4_)_2_ square nanoplates as an example, time-dependent morphology evolution and XRD analysis are carried out to disclose the growth mechanism of NaGd(MoO_4_)_2_ nanocrystals. The SEM images and XRD patterns of the products obtained for different reaction time (0, 0.5, 1, 3 and 6 h) with 1.25 ml oleic acid and 10 mmol Na_2_MoO_4_ in the initial precursor solutions are presented in Supplementary Fig. S6. Amorphous poor-crystalline precursors were formed in the initial stage before hydrothermal reaction, which can be confirmed by the corresponding SEM images and XRD patterns of the products obtained at 0 h. When the reaction time is prolonged to 0.5 h, some small particles appear in the SEM image (Supplementary Fig. S6b) and a small peak emerges in the XRD pattern, which means crystal nuclei are gradually formed as the hydrothermal reaction proceed. Square nanoplates are found when the reaction time is prolonged to 1 h and some small ones are also found in the SEM image (Supplementary Fig. S6c). Meanwhile the diffraction peaks in the XRD patterns of the products become sharper and stronger, and the peaks fit well with the pure tetragonal phase NaGd(MoO_4_)_2_. This indicates that the nuclei grow bigger first to form rudiments of NaGd(MoO_4_)_2_ nanocrystals with the morphology of square nanoplates under hydrothermal conditions in the presence of oleic acid. Then some of the rudimental crystals grow even bigger. By and large, the nanocrystals in this stage are not well developed, since some square nanoplates with smaller size are always observed in the SEM image (Supplementary Fig. S6c). With further increasing reaction time, the rudimental NaGd(MoO_4_)_2_ square plates grow bigger and bigger, and the smaller ones disappear gradually in the meantime (Supplementary Fig. S6c–e). This can be deemed as the ripening process of the NaGd(MoO_4_)_2_ nanocrystals. In this stage, large square nanoplates develop even bigger at the expense of smaller ones, driven by the thermodynamic minimization of the surface energies of the nanocrystals. This phenomenon is often observed in the synthesis process of nanocrystals, and universally known as Ostwald ripening^45^. Eventually, uniform and well-crystallized NaGd(MoO_4_)_2_ nanocrystals with regular morphology are formed at the end of the ripening process. A possible growth mechanism is proposed based on the morphology evolution as follows. First the precursor is converted to NaGd(MoO_4_)_2_ nuclei in the nucleation stage under hydrothermal conditions. Subsequently, the NaGd(MoO_4_)_2_ nuclei grow to rudimental NaGd(MoO_4_)_2_ nanocrystals, followed by the Ostwald ripening process until well-crystallized NaGd(MoO_4_)_2_ nanocrystals are formed. In brief, NaGd(MoO_4_)_2_ nanocrystals are formed through “Nucleation → Crystallization → Ostwald ripening” growth process.

**Figure 4:**
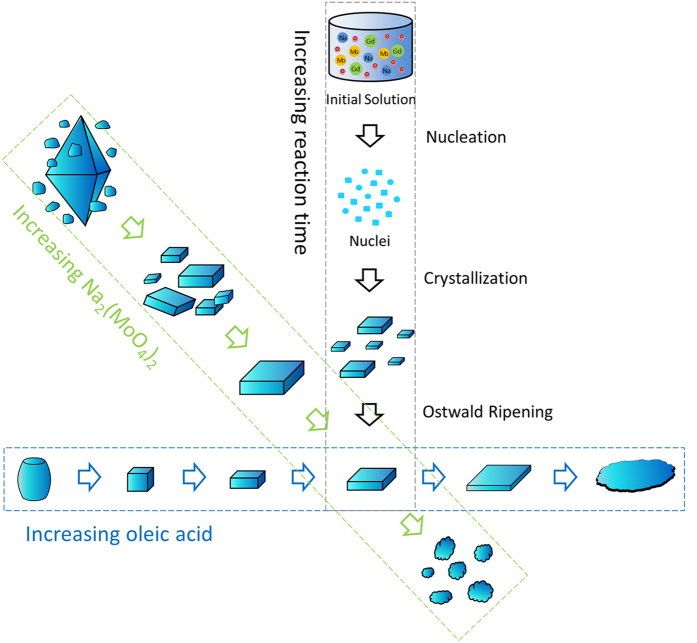
Schematic illustration of the effects of Na_2_MoO_4_ and oleic acid on the formation of NaGd(MoO_4_)_2_ nanocrystals with diverse morphologies and the growth mechanism of NaGd(MoO_4_)_2_ square nanoplates.

From the above analysis, it can be seen that the amounts of Na_2_MoO_4_ and oleic acid have significant effects on the formation of the NaGd(MoO_4_)_2_ nanocrystals, and the NaGd(MoO_4_)_2_ nanocrystals are formed step by step with increasing reaction time under hydrothermal conditions in the growth process. The effects of Na_2_MoO_4_ and oleic acid on the formation of NaGd(MoO_4_)_2_ nanocrystals with diverse morphologies, and the growth mechanism of NaGd(MoO_4_)_2_ square nanoplates is summarized and illustrated schematically in Fig. 4.

### Photoluminescence and thermometric properties of NaGd(MoO_4_)_2_: nanocrystals upon lanthanide (Eu^3+^, Yb^3+^/Er^3+^ and Yb^3+^/Tm^3+^) doping

#### Morphology-dependent down-shifting photoluminescence of NaGd(MoO_4_)_2_: Eu^3+^ nanocrystals

**Figure 5:**
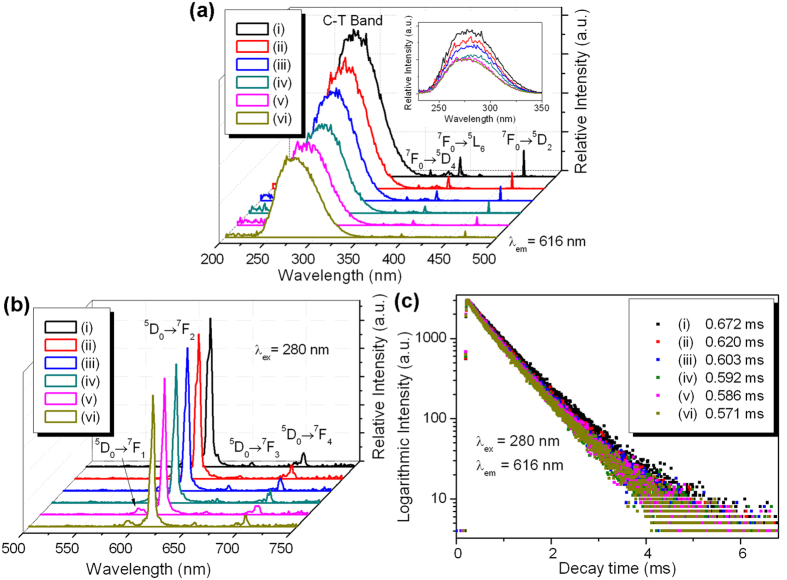
Photoluminescence excitation spectra (**a**), emission spectra (**b**) and decay profiles (**c**) of NaGd(MoO_4_)_2_: 5% Eu^3+^ nanocrystals with different morphologies: (i) nanocubes, (ii–vi) nanoplates with decreasing thicknesses. Inset of (**a**) shows the spectra of the C-T bands.

NaGd(MoO_4_)_2_: 5% Eu^3+^ nanocrystals with the morphologies of nanocubes and square nanoplates with different thicknesses (corresponding to the morphologies shown in Fig. 3b–g) are synthesized, and their morphology-dependent down-shifting photoluminescence properties are investigated in detail. Figure 5a shows the photoluminescence excitation spectra of NaGd(MoO_4_)_2_: 5% Eu^3+^ nanocrystals monitoring at 616 nm. Broad and intense excitation bands lie in the range from 235 to 350 nm, which are referred to charge transfer (C-T) absorption corresponding to the electron transfer from 2p orbit of O^2−^ to 5d orbit of Mo^6+^ within the MoO_4_^2−^ group in the host molybdate. The full widths at half-maximum (FWHM) of the C-T bands are all about 55 nm. These broad and intense C-T excitation bands indicate that the dopant Eu^3+^ in NaGd(MoO_4_)_2_ nanocrystals can be excited efficiently by ultraviolet light radiation around 280 nm. Sharp peaks centered at 362, 395 and 465 nm are observed for all spectra shown in Fig. 5a. These peaks are attributed to the characteristic 4*f *→* *4*f* transitions (^7^F_0_ → ^5^D_4_, ^7^F_0_ → ^5^L_6_ and ^7^F_0_ → ^5^D_2_) of Eu^3+^. The asymmetric C-T band can be fitted by two Gaussian peaks, and the fitting curves for spectrum (i) and (vi) in Fig. 5a are depicted in Supplementary Figs S7 and S8. A blue-shift of more than 2 nm in the C-T bands from spectrum (i) to (vi) is observed in the fitting curves. The nanocrystals with a smaller thickness will have a larger energy gap due to quantum confinement effect. The charge transfer band, which is related to the bandgap of NaGd(MoO_4_)_2_ host, is thus shifted towards the higher energy side. On the other hand, the blue-shift of the C-T bands indicate that the bonding energy between the central Mo^6+^ and the ligand O^2−^ becomes stronger with the morphology varying from nanocubes to thin nanoplates. The intensities of C-T bands and other intrinsic peaks of Eu^3+^ in excitation spectrum decrease gradually, which result from luminescent quenching effect.

Figure 5b shows the photoluminescence emission spectra of NaGd(MoO_4_)_2_: 5% Eu^3+^ nanocrystals under excitation of C-T band at 280 nm. The spectra for different morphologies are similar in shape, in which four emission peaks at 592, 616, 655, and 703 nm are associated with ^5^D_0_ → ^7^F_*J*_ (*J* = 1, 2, 3, 4) transitions of Eu^3+^. The sharp and intense red emission lines at 616 nm (^5^D_0_ → ^7^F_2_ transition) suggest that the Eu^3+^ dopant ions occupy the sites without inversion symmetry in the host NaGd(MoO_4_)_2_ nanocrystals. What’s more, the emission intensity decreases slightly from spectrum (i) to (vi) due to the decreased radiative transition probability caused by surface quenching effect. Compared with the nanocubes, the thin nanoplates are smaller in size and have a larger surface-to-volume ratio. The energy of activator (Eu^3+^) may be trapped by surface defects, ligands and other quenchers which leads to enhanced surface quenching effect^46^. The luminescence dynamics of NaGd(MoO_4_)_2_: 5% Eu^3+^ nanocrystals at 616 nm for different morphologies are investigated, as shown in Fig. 5c. All the curves show single-exponential decay and can be well-fitted by a single-exponential function *I* = *I*_0_ exp(−*t*/*τ*), where *I*_0_ is the luminescence intensity at *t* = 0, *τ* is the lifetime. The lifetimes of Eu^3+^ are determined to be 0.672, 0.620, 0.603, 0.592, 0.586 and 0.571 ms for different morphologies from nanocubes to thin square nanoplates, respectively. The lifetime of an excited state depends on the depopulation (radiative or nonradiative transitions) probability of electrons from this excited state. Due to the surface quenching effect, the energy in the upper excited state of Er^3+^ easily migrates to the surface and is trapped by the surface defects, ligands or other quenchers, which increases the nonradiative transition probability and therefore reduces the lifetime of the excited state. So the thin square nanoplates with the smallest size and the largest surface-to-volume ratio have the lowest lifetime.

#### Upconversion photoluminescence of NaGd(MoO_4_)_2_: Yb^3+^/Er^3+^ and Yb^3+^/Tm^3+^ thin square nanoplates

**Figure 6:**
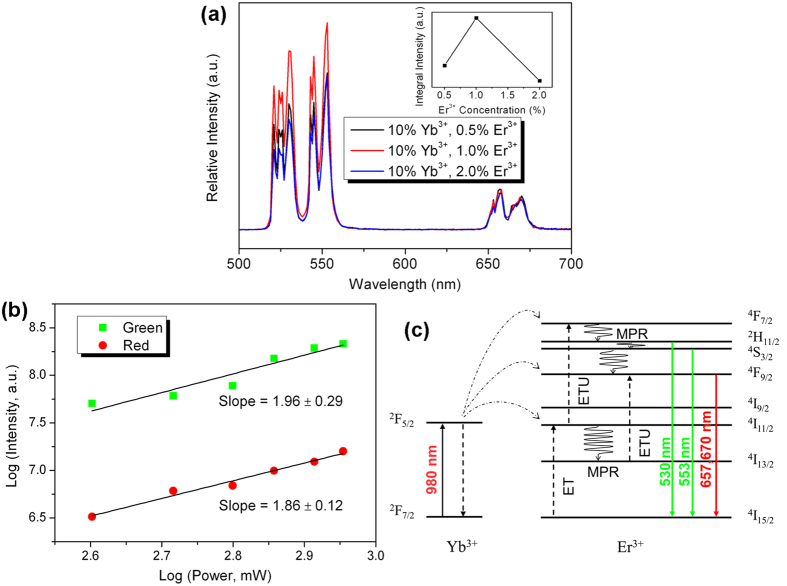
(**a**) Upconversion luminescence spectra of NaGd(MoO_4_)_2_: Yb^3+^/Er^3+^ nanoplates under excitation of 980 nm, and inset of (**a**) shows the dependence of emission intensities on Er^3+^ concentrations; (**b**) Double logarithmic plots of green and red upconversion emission intensities versus pump powers and linear fitting curves of NaGd(MoO_4_)_2_: 10% Yb^3+^/1% Er^3+^ nanoplates; (**c**) Upconversion mechanism and energy level scheme of NaGd(MoO_4_)_2_: Yb^3+^/Er^3+^ nanoplates excited at 980 nm.

Upconversion luminescence properties of NaGd(MoO_4_)_2_: Yb^3+^/Er^3+^, Yb^3+^/Tm^3+^ thin nanoplates are investigated. Figure 6a presents the upconversion luminescence spectra of NaGd(MoO_4_)_2_: Yb^3+^/Er^3+^ nanoplates with fixed Yb^3+^ concentration (10%) and different Er^3+^ concentrations (0.5%, 1% and 2%) under 980 nm excitation. Intense green luminescence peaks centered at 530 and 553 nm, and red emission peaks at 657 and 670 nm are observed, which are both characteristic intra-configurational 4*f* → 4*f* transitions of Er^3+^. The two green emission peaks at 530 and 553 nm are ascribed to the transitions ^2^H_11/2_ → ^4^I_15/2_ and ^4^S_3/2_ → ^4^I_15/2_ of Er^3+^, respectively. The red emission peaks correspond to the transition ^4^F_9/2_ → ^4^I_15/2_ of Er^3+^. Both the green and red upconversion emissions are split into several subpeaks due to Stark splitting of the upper energy levels. The integral emission intensities of the three samples are depicted in the inset of Fig. 6a. The sample with Er^3+^ doping concentration of 1% possesses the highest integral emission intensity, indicating the optimal Er^3+^ doping concentration is 1%. At higher Er^3+^ doping concentration the upconversion luminescence becomes less efficient, owing to the concentration quenching effect and the cross relaxation between Er^3+^.

To ascertain the upconversion energy transfer mechanism, investigation of power dependence of upconversion emissions is performed. The double logarithmic plots of green and red upconversion emission intensities versus pump powers for the NaGd(MoO_4_)_2_: 10% Yb^3+^/1% Er^3+^ nanoplates are depicted in Fig. 6b, together with the linear fitting curves. Generally, the number of photons involved in the upconversion process may be inferred from the slopes of the plots^47^. The slope value of the fitting curves for green and red upconversion emissions is 1.96 and 1.86 respectively, revealing two photons are involved in both green and red upconversion processes.

The proposed upconversion mechanism based on the energy level scheme and power dependence of upconversion luminescence is schematically shown in Fig. 6c. Yb^3+^ ions act as sensitizer to absorb energy of 980 nm excitation light and transfer it to activator Er^3+^ ions. Electrons in the ground state (^4^I_15/2_) of Er^3+^ can be excited to ^4^I_11/2_ state through energy transfer (ET) process from Yb^3+^, and subsequently excited to ^4^F_7/2_ state through energy transfer upconversion (ETU) process. The states ^2^H_11/2_ and ^4^S_3/2_ can be populated by means of nonradiative multiphonon relaxation (MPR) form the state ^4^F_7/2_. Radiative transitions from ^2^H_11/2_/^4^S_3/2_ to the ground state of Er^3+^ generate green upconversion emission. For red upconversion emission, there are two ways to populate the upper excited state ^4^F_9/2_: MPR process from state ^4^S_3/2_ and ETU process from state ^4^I_13/2_. Then red upconversion emission can be expected by radiative transition from the populated state ^4^F_9/2_ to the ground state.

**Figure 7:**
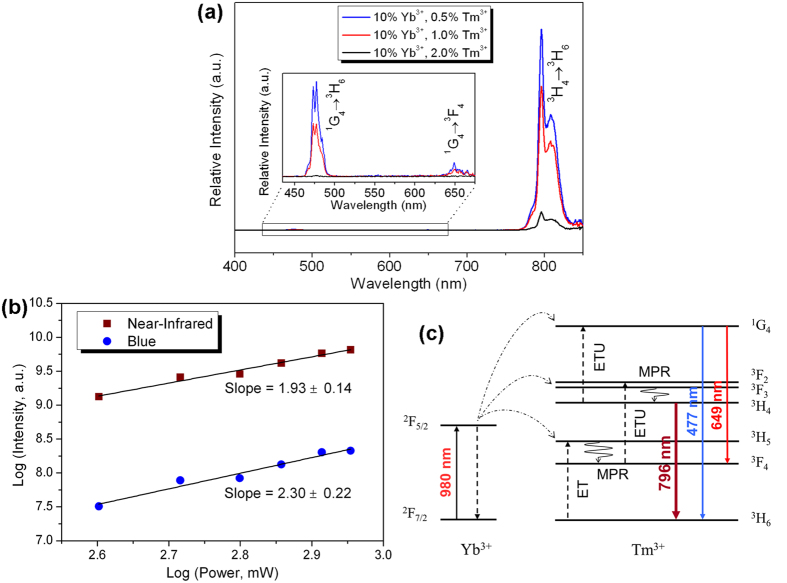
(**a**) Upconversion luminescence spectra of NaGd(MoO_4_)_2_: Yb^3+^/Tm^3+^ nanoplates under excitation of 980 nm, and Inset of (**a**) shows the magnified spectrum in the visible wave range from 435 to 675 nm; (**b**) Double logarithmic plots of near-infrared upconversion emission intensities versus pump powers and linear fitting curves of NaGd(MoO_4_)_2_: 10% Yb^3+^/0.5% Tm^3+^ nanoplates; (**c**) Upconversion mechanism and energy level scheme of NaGd(MoO_4_)_2_: Yb^3+^/Tm^3+^ nanoplates excited at 980 nm.

Figure 7a presents the upconversion luminescence spectra of NaGd(MoO_4_)_2_: Yb^3+^/Tm^3+^ nanoplates with fixed Yb^3+^ concentration (10%) and different Tm^3+^ concentrations (0.5%, 1% and 2%) under 980 nm excitation. Intense emission peaks at 796 nm in the infrared wave range corresponding to the transition ^3^H_4_ → ^3^H_6_ of Tm^3+^ are observed in all the three spectra. All the three samples exhibit nearly pure near-infrared upconversion luminescence. The upconversion emission intensity of the NaGd(MoO_4_)_2_: Yb^3+^/Tm^3+^ samples decreases with increasing Tm^3+^ concentration, which is caused by the concentration quenching effect. The sample doped with 10% Yb^3+^/0.5% Tm^3+^ possesses the most intense emission intensity. Some relatively weak emission peaks can also be observed in the visible region of the magnified spectra for the samples doped with 0.5% and 1% Tm^3+^ (shown in inset of Fig. 7a). Blue emission peaks at 477 nm and red emission peaks at 649 nm are ascribed to transitions ^1^G_4_ → ^3^H_6_ and ^1^G_4_ → ^3^F_4_ of Tm^3+^. Since the emission peaks at 796 nm locate in the invisible wave range, the upconversion luminescence appears blue in color to the naked eye.

Figure 7b depicts the double logarithmic plots of near-infrared upconversion emission intensities versus pump powers for the NaGd(MoO_4_)_2_: 10% Yb^3+^/0.5% Tm^3+^ nanoplates. The slope value of the fitting curves is 1.93 for near-infrared upconversion emissions and 2.30 for near-infrared emission, suggesting that the near-infrared upconversion luminescence belongs to two-photon process, while the blue one involve three-photon absorption. Figure 7c shows the proposed upconversion mechanism and the energy level scheme of NaGd(MoO_4_)_2_: Yb^3+^/Tm^3+^. There are energy mismatches between the transitions within Yb^3+^ (^2^F_5/2_ → ^2^F_7/2_) and Tm^3+^ (^3^H_6_ → ^3^H_5_, ^3^F_4_ → ^3^F_2_, and ^3^H_4_ → ^1^G_4_). So the energy transfer between Yb^3+^ and Tm^3+^ needs the assistance of phonons of the host. State ^3^H_5_ is populated from ^3^H_6_ by phonon assisted ET process. Electrons in state ^3^H_5_ can relax to state ^3^F_4_ through the MPR process. The phonon assisted ETU process will populate the state ^3^F_2, 3_ from state ^3^F_4_. Electrons in the state ^3^F_2, 3_ can relax to state ^3^H_4_, from which electrons relax radiatively to the ground state generating dominated near-infrared upconversion emission (796 nm). State ^1^G_4_ might be populated by another phonon assisted ETU process from ^3^H_4_ to ^1^G_4_. Electrons in ^1^G_4_ state can decay radiatively to either state ^3^F_4_ or state ^3^H_6_, which will cause the blue (477 nm) or red (649 nm) upconversion emissions. For the ET and ETU processes ^3^H_6_ → ^3^H_5_, ^3^F_4_ → ^3^F_2_, and ^3^H_4_ → ^1^G_4_, the number of phonons needed in a similar host NaGd(WO_4_)_2_ is about 2, 3 and 5, respectively^48^. The more the number of phonons is, the lower the probability of energy transfer is. Therefore, it is speculated that the ETU process ^3^H_4_ → ^1^G_4_ hardly occurs and the electrons in the ^3^H_4_ state are likely to relax radiatively to the ground state rather than to be excited to ^1^G_4_ state through phonon assisted ETU process in our case. This explains why the infrared upconversion luminescence is quite intense compared with the visible upconversion luminescence.

#### Thermometric properties of NaGd(MoO_4_)_2_: Yb^3+^/Er^3+^ thin square nanoplates

**Figure 8:**
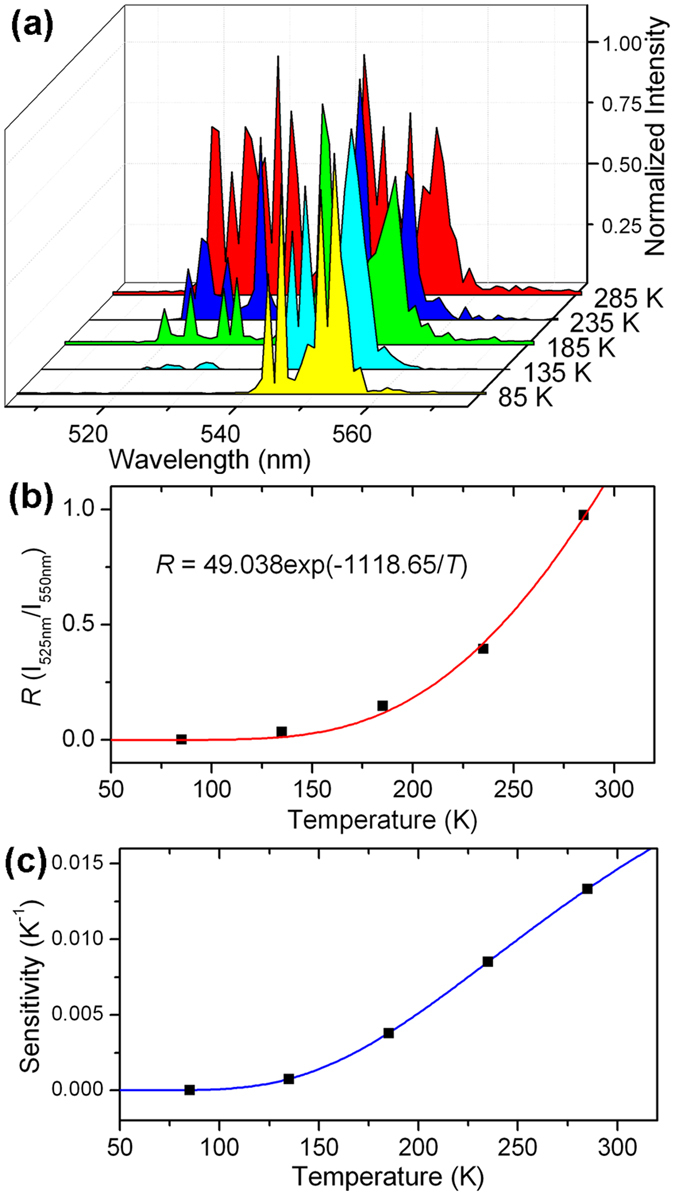
(**a**) Temperature-dependent upconversion luminescence spectra (normalized to 1 at the maximum emission value) of NaGd(MoO_4_)_2_: 10% Yb^3+^/1% Er^3+^ square plate nanocrystals from 85 to 285 K; (**b**,**c**) Dependence of intensity ratio *R* and thermometric sensitivity of NaGd(MoO_4_)_2_: 10% Yb^3+^/1% Er^3+^ square plate nanocrystals on temperature. The sensitivity at 285 K is 0.01333 K^−1^.

Since the emission intensity ratio from two thermally coupled energy levels of lanthanide ions is sensitive to ambient temperature, optical thermometry can be realized in Ln^3+^ doped upconversion nanocrystals based on the temperature-dependent upconversion luminescence^49^. Adjacent thermally coupled energy levels ^2^H_11/2_ and ^4^S_3/2_ of Er^3+^ follow a Boltzmann-type population distribution, and are employed to investigate thermometric properties of NaGd(MoO_4_)_2_: 10% Yb^3+^/1% Er^3+^ nanoplate crystals. Figure 8a shows the temperature-dependent upconversion luminescence spectra (normalized to 1 at the maximum emission value) from 85 to 285 K under excitation of 980 nm. The upconversion luminescence intensity ratio *R* (I_525nm_/I_550nm_) of the two green emission bands increases evidently with increasing ambient temperature.

The intensity ratio *R* can be expressed as:


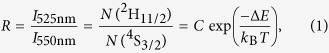


where *N* is the population number of the energy level, *C* is a proportionality constant (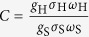, where *g, σ, ω* are degeneracy, emission cross-section and angular frequency of radiative transitions from the ^2^H_11/2_ and ^4^S_3/2_ levels to the ground level ^4^I_15/2_), Δ*Ε* is the energy gap between the ^2^H_11/2_ and ^4^S_3/2_ levels, *k*_B_ is the Boltzmann constant, and *T* is the absolute temperature. The dependence of upconversion luminescence intensity ratio *R* with the temperature is plotted in Fig. 8b. The intensity ratio *R* varies from 0.0006 to 0.9752 with the temperature increasing from 85 to 285 K. The fitting curve is also presented according to the above equation, which matches well with the experimental data. Δ*Ε* can be further calculated to be 777.45 cm^−1^ from the fitting results, which is very close to the experimental energy gap between the two levels.

The thermometric sensitivity *S* is defined as the rate of change of *R*(*T*) as follows,


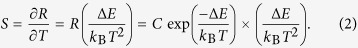


The calculated sensitivity is plotted as a function of absolute temperature in Fig. 8c. The thermometric sensitivity of NaGd(MoO_4_)_2_: Yb^3+^/Er^3+^ nanocrystals increases with increasing temperature in the temperature range of measurement. Compared with the square plate NaGd(MoO_4_)_2_: 10% Yb^3+^/1% Er^3+^ microcrystals synthesized as previously reported by us (temperature-dependent upconversion luminescence and thermometric sensitivity are shown in Supplementary Figs S9 and S10), the NaGd(MoO_4_)_2_: 10% Yb^3+^/1% Er^3+^ nanocrystals possess a more sensitive thermometric property. The maximum value of sensitivity of the NaGd(MoO_4_)_2_: Yb^3+^/Er^3+^ nanocrystals is 0.01333 K^−1^ at 285 K, which is higher than reported values of many other Yb^3+^/Er^3+^ doped materials in a similar temperature range (around 300 K)^23^,^49^,^50^,^51^,^52^,^53^,^54^,^55^.

From the fitted equations for luminescence intensity ratio (*R*)-Temperature curves for nanocrystals and microcrystals (shown in Fig. 8b and Supplementary Fig. S10a), it is found that, compared with that for microcrystals, both Δ*E* and the proportionality constant *C* increase in Equation (1) for nanocrystals. The larger Δ*E* (energy gap between ^2^H_11/2_ and ^4^S_3/2_) is, the lower the MPR probability from state ^2^H_11/2_ to state ^4^S_3/2_ is, which causes the enhancement of the radiative transition probability of the state ^2^H_11/2_. The enhanced radiative transition probability from state ^2^H_11/2_ causes the increase in the value of the emission cross-section of radiative transitions from the ^2^H_11/2_ (*σ*_H_). Considering 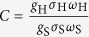, the value of *C* for nanocrystals increases, which leads to the enhanced sensitivity as defined in Equation (2). This is why nanocrystals have a more sensitive response to temperature, compared with microcrystals.

## Conclusion

Pure tetragonal phase, uniform and well-crystallized NaGd(MoO_4_)_2_ nanocrystals with diverse regular morphologies can be selectively synthesized *via* oleic acid-mediated hydrothermal synthesis method by simply tuning the contents of oleic acid in the precursor solution. (MoO_4_)^2−^ ions will be preferentially adsorbed on the {001} facets of tetragonal NaGd(MoO_4_)_2_, which have a higher packing density of Gd^3+^/Na^+^ ions (0.0364 Å^−2^). Thus, appropriate amount of Na_2_MoO_4_ in the precursor solution favor the formation of NaGd(MoO_4_)_2_ nanocrystals with regular morphology. Since oleic acid in the hydrothermal solution helps to control the growth rate of the nanocrystals, especially along [001] directions of tetragonal NaGd(MoO_4_)_2_, the amount of oleic acid plays a key role in the morphology-controlled synthesis of NaGd(MoO_4_)_2_ nanocrystals. The morphology of the as-synthesized NaGd(MoO_4_)_2_ nanocrystals evolves in the sequence from nanocylinders, nanocubes, square nanoplates to irregular nanoflakes with increasing oleic acid content. Time-dependent morphology evolution and XRD analysis of the products suggest that the growth of NaGd(MoO_4_)_2_ nanocrystals is governed by a “Nucleation → Crystallization → Ostwald ripening” growth mechanism. Investigation of down-shifting photoluminescence properties confirm that lanthanide dopant in NaGd(MoO_4_)_2_ host nanocrystals can be excited efficiently by broad band ultraviolet light through charge transfer absorption (around 280 nm). Due to quantum confinement effect and stronger bonding energy between the Mo^6+^ and ligand O^2−^, the charge transfer band has a slight blue-shift, and the intensity is decreased with the morphology of the nanocrystals varying from nanocubes to thin nanoplates. As a result of surface quenching effect, both the down-shifting emission intensity and lifetime of the Eu^3+^ doped nanocrystals decrease gradually from nanocubes to thin square nanoplates. On the basis of energy level scheme and pump power dependence of upconversion emissions, the mechanisms for upconversion photoluminescence of NaGd(MoO_4_)_2_: Yb^3+^/Er^3+^, Yb^3+^/Tm^3+^ nanocrystals are proposed. Two-photon process accounts for both the visible (green and red) upconversion emissions of NaGd(MoO_4_)_2_: Yb^3+^/Er^3+^ nanocrystals and the near-infrared upconversion emission of NaGd(MoO_4_)_2_: Yb^3+^/Tm^3+^ nanocrystals. While the blue upconversion emission of NaGd(MoO_4_)_2_: Yb^3+^/Tm^3+^ nanocrystals involves three-photon absorption. Lower probability of phonon assisted ETU process ^3^H_4_ → ^1^G_4_ of Tm^3+^ lead to nearly pure near-infrared upconversion luminescence of NaGd(MoO_4_)_2_: Yb^3+^/Tm^3+^ nanocrystals. NaGd(MoO_4_)_2_: Yb^3+^/Er^3+^ nanocrystals exhibit excellent thermometric properties with a relatively high sensitivity (0.01333 K^−1^ at 285 K). NaGd(MoO_4_)_2_ nanocrystals have a more sensitive response to temperature compared with microcrystals. Investigations of photoluminescence and thermometric properties manifest that NaGd(MoO_4_)_2_ nanocrystals are promising candidates for luminescent hosts in luminescent imaging, temperature sensing, color display and other tremendous down-shifting/upconversion applications.

## Methods

### Chemicals

Gd_2_O_3_ (99.99%), Eu_2_O_3_ (99.99%), Yb_2_O_3_ (99.99%), Er_2_O_3_ (99.99%), Tm_2_O_3_ (99.99%), sodium molybdate dihydrate (Na_2_MoO_4_·2H_2_O, analytical grade), oleic acid (C_18_H_34_O_2_, analytical grade), nitric acid (HNO_3_, analytical grade) and ethanol (analytical grade).

### Preparation of lanthanide nitrates

Lanthanide nitrates (Gd(NO_3_)_3_, Eu(NO_3_)_3_, Yb(NO_3_)_3_, Er(NO_3_)_3_ and Tm(NO_3_)_3_) were prepared by dissolving appropriate amounts of Gd_2_O_3_, Eu_2_O_3_, Yb_2_O_3_, Er_2_O_3_ and Tm_2_O_3_, in diluted nitric acid respectively, under vigorous stirring and heating in water bath until evaporated. Lanthanide nitrate solutions were prepared by dissolving the corresponding lanthanide nitrates in deionized water.

### Synthesis of NaGd(MoO_4_)_2_ and NaGd(MoO_4_)_2_: Eu^3+^, Yb^3+^/Er^3+^ and Yb^3+^/Tm^3+^ nanocrystals

A predetermined amount of oleic acid was added into 10 ml ethanol. After vigorous stirring for 30 min, 0.5 ml Gd(NO_3_)_3_ solution (0.5 mmol) and a certain amount of Na_2_MoO_4_ solution were added into the above solution under continuous stirring. After additional agitation for 1 h, the as-obtained translucent precursor solution (total volume 41 ml) was transferred into a 60 ml Teflon-lined stainless steel autoclave, which was then sealed and maintained at 180 °C for 12 h. The final precipitate products were collected by centrifugation, washed several times with deionized water and ethanol, and dried at 50 °C for 5 h in air.

NaGd(MoO_4_)_2_: Eu^3+^, Yb^3+^/Er^3+^, Yb^3+^/Tm^3+^nanocrystals were synthesized following a similar procedure except for introducing the proper amount of corresponding lanthanide nitrates to the precursor solution as described above.

### Characterization

Powder X-ray diffraction (XRD) was performed on a Rigaku Smartlab diffractometer with Cu Kα radiation at a scanning rate of 10° min^−1^. Scanning electron microscope (SEM, FEI Quanta 400F) and transmission electron microscope (TEM, FEI Tecnai G2 F30) were employed for the observation of the morphology. Energy dispersive X-ray spectroscopy (EDS) data were obtained using the SEM equipped with the energy dispersive X-ray spectrometer. TEM images, high-resolution TEM (HRTEM) images and selected-area electron diffraction (SAED) patterns were performed at an accelerating voltage of 300 kV. Fourier transform infrared (FTIR) spectra were obtained in transmission mode on a Bruker Equinox 55 FTIR spectrometer with the samples sandwiched between two KBr plates. Photoluminescence excitation and emission spectra were recorded on an Edinburgh FLSP920 spectrometer equipped with a 980 nm diode laser, a 450 W continuous xenon lamp and a 60 W microsecond flash lamp as excitation sources and a R928 red-sensitive photomultiplier tube as detector. The samples were annealed at 500 °C for 1 h prior to upconversion luminescence measurements. All the measurements were performed at room temperature except for the thermometric upconversion photoluminescence.

## Additional Information

**How to cite this article**: Li, A. *et al*. NaGd(MoO_4_)_2_ nanocrystals with diverse morphologies: controlled synthesis, growth mechanism, photoluminescence and thermometric properties. *Sci. Rep.* **6**, 31366; doi: 10.1038/srep31366 (2016).

## Supplementary Material

Supplementary Information
