# Supplementary material for: NaGd(MoO4)2 nanocrystals with diverse morphologies: controlled synthesis, growth mechanism, photoluminescence and thermometric properties
Source: Sci Rep. 2016 Aug 10;6:31366. doi: 10.1038/srep31366 (PMC4979211; doi:10.1038/srep31366)
Supplement: Supplementary Information [file srep31366-s1.doc]

*Supplementary Information*

**NaGd(MoO_4_)_2_ nanocrystals with diverse morphologies: controlled synthesis, growth mechanism, photoluminescence and thermometric properties**

Anming Li^1,2^, Dekang Xu^1^, Hao Lin^1^, Shenghong Yang^1^, Yuanzhi Shao^1^ and Yueli Zhang^1^,*

^1^State Key Laboratory of Optoelectronic Materials and Technologies, School of Materials Science and Engineering / School of Physics, Sun Yat-sen University, Guangzhou, 510275, China.

^2^Institute of Optoelectronic Engineering, Department of Optoelectronic Engineering, Jinan University, Guangzhou, 510632, China.

[^*^stszyl@mail.sysu.edu.cn](mailto:*stszyl@mail.sysu.edu.cn).


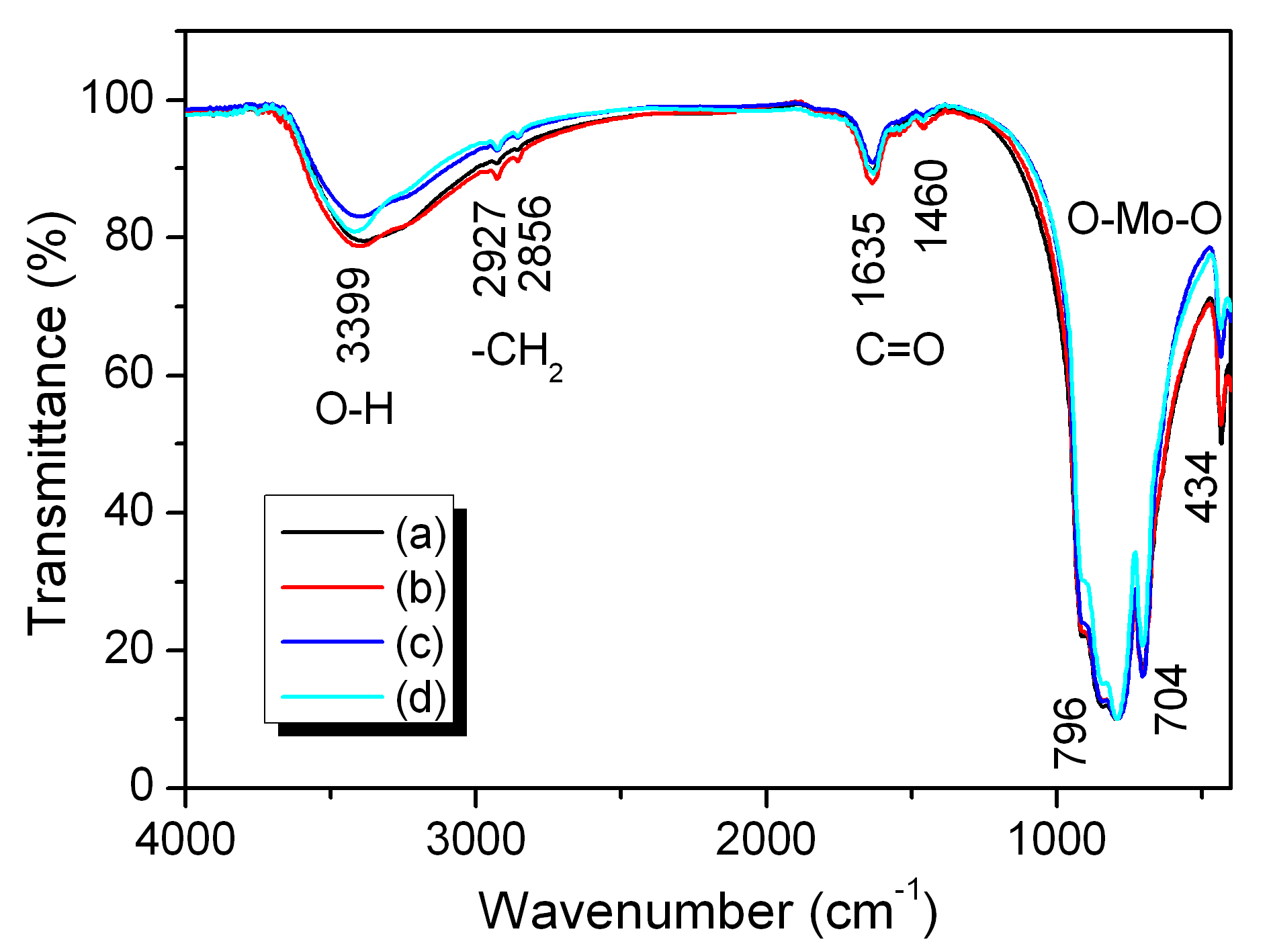


**Fig. S1** FTIR spectra of the NaGd(MoO_4_)_2_ nanocrystals with four typical morphologies (a–d) corresponding to four typical morphologies shown in Fig. 2a–d.

**
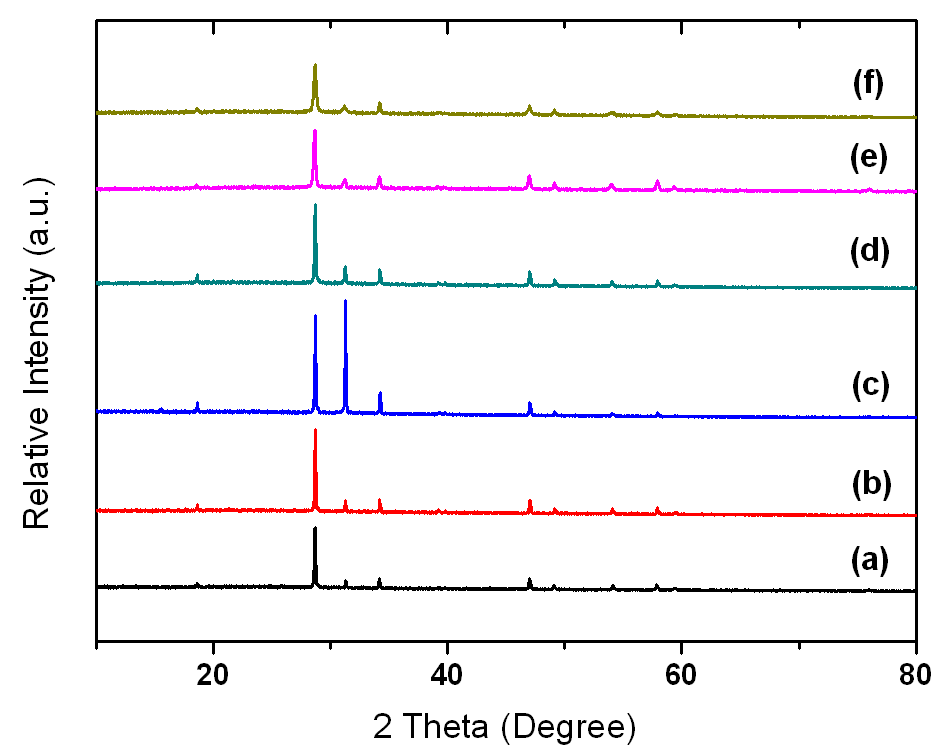
**

**Fig. S2** XRD patterns of the NaGd(MoO_4_)_2_ nanocrystals synthesized with different contents of Na_2_MoO_4_: (a) 2 mmol, (b) 4 mmol, (c) 6 mmol, (d) 8 mmol, (e) 10 mmol, (f) 12 mmol.

**
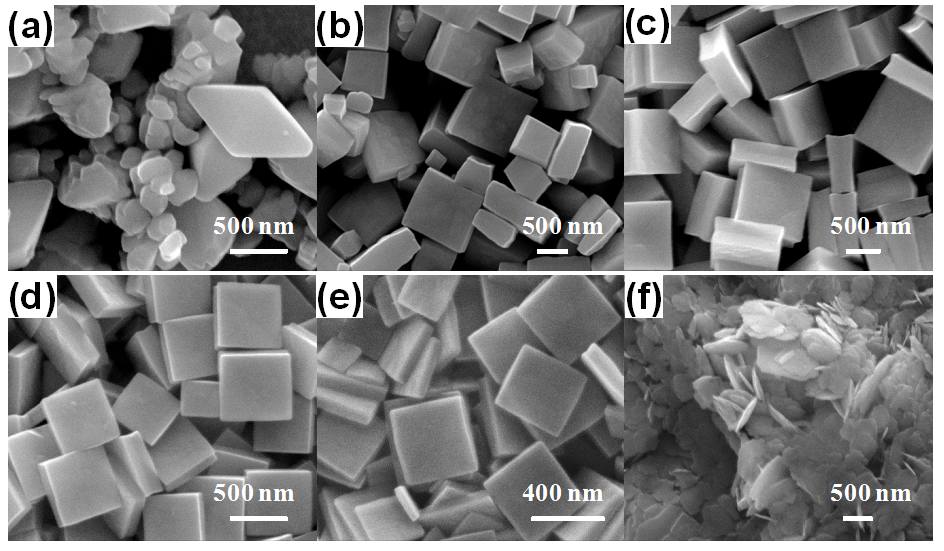
**

**Fig. S3** SEM images of NaGd(MoO_4_)_2_ nanocrystals synthesized with different contents of Na_2_MoO_4_: (a) 2 mmol, (b) 4 mmol, (c) 6 mmol, (d) 8 mmol, (e) 10 mmol, (f) 12 mmol.

**
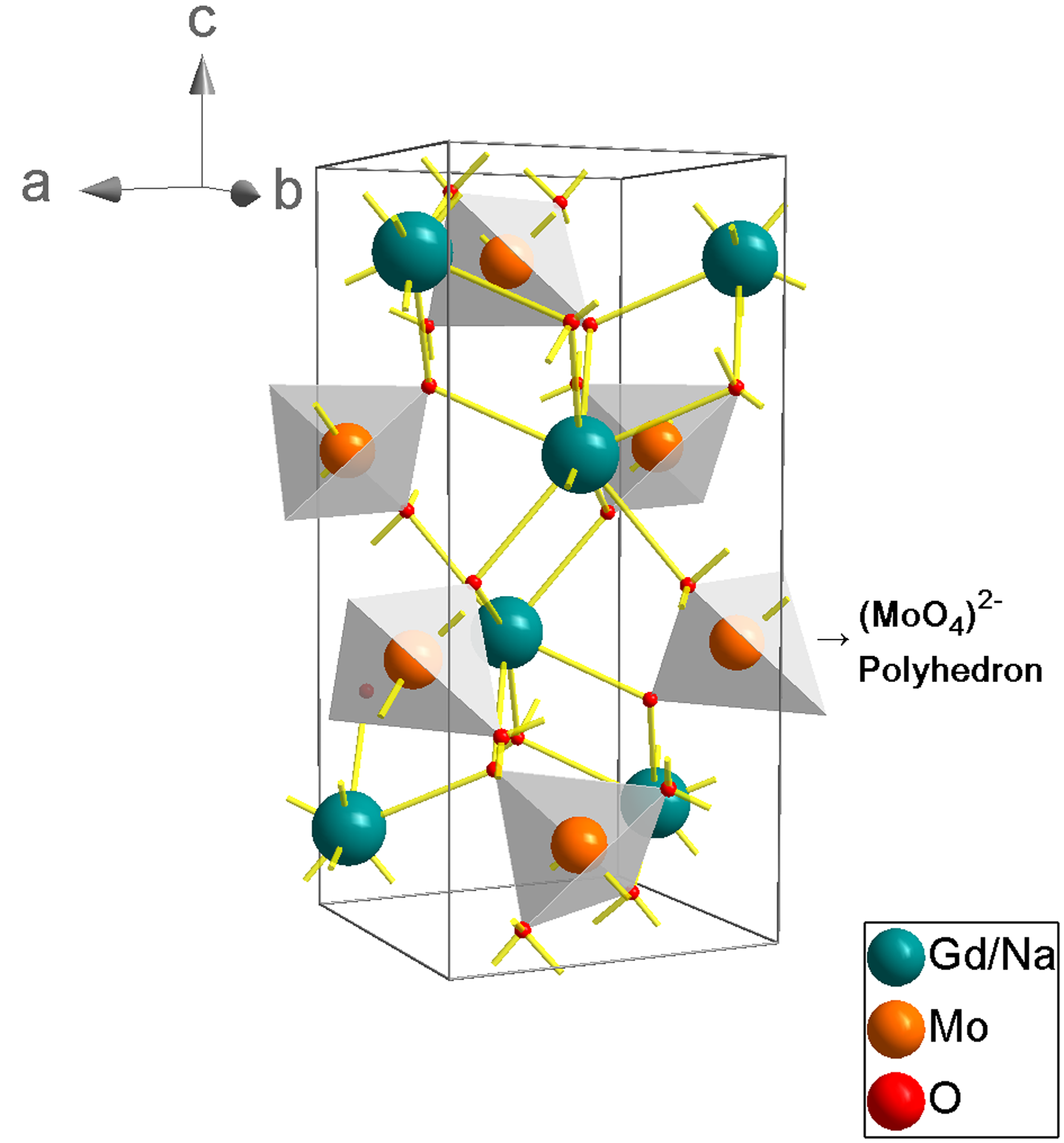
**

**Fig. S4** Schematic crystal structure of the NaGd(MoO_4_)_2_ with one unit cell.


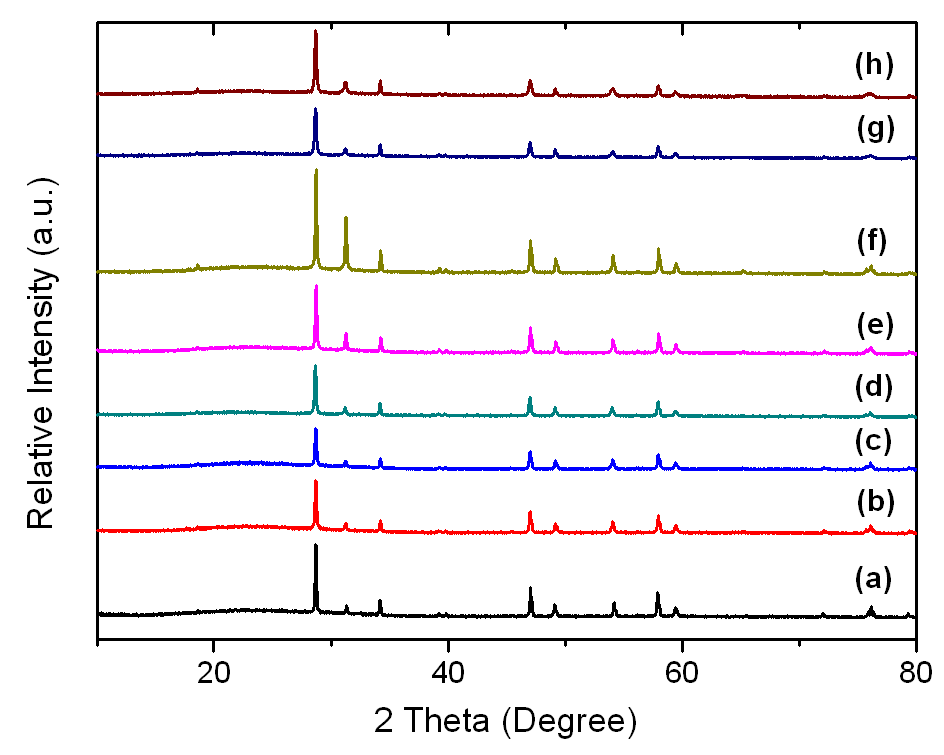


**Fig. S5** XRD patterns of the NaGd(MoO_4_)_2_ nanocrystals synthesized with different contents of oleic acid, (a) 0 ml, (b) 0.25 ml, (c) 0.5 ml, (d) 0.75 ml, (e) 1 ml, (f) 1.25 ml, (g) 1.5 ml, (h) 1.75 ml.


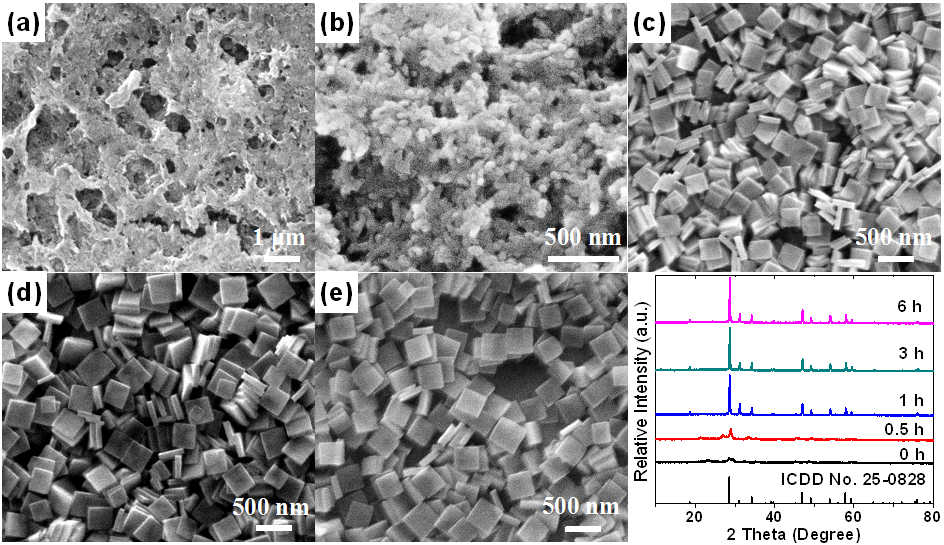


**Fig. S6** SEM images and XRD patterns of the products obtained at different reaction time: (a) 0 h, (b) 0.5 h, (c) 1 h, (d) 3 h, (e) 6 h.


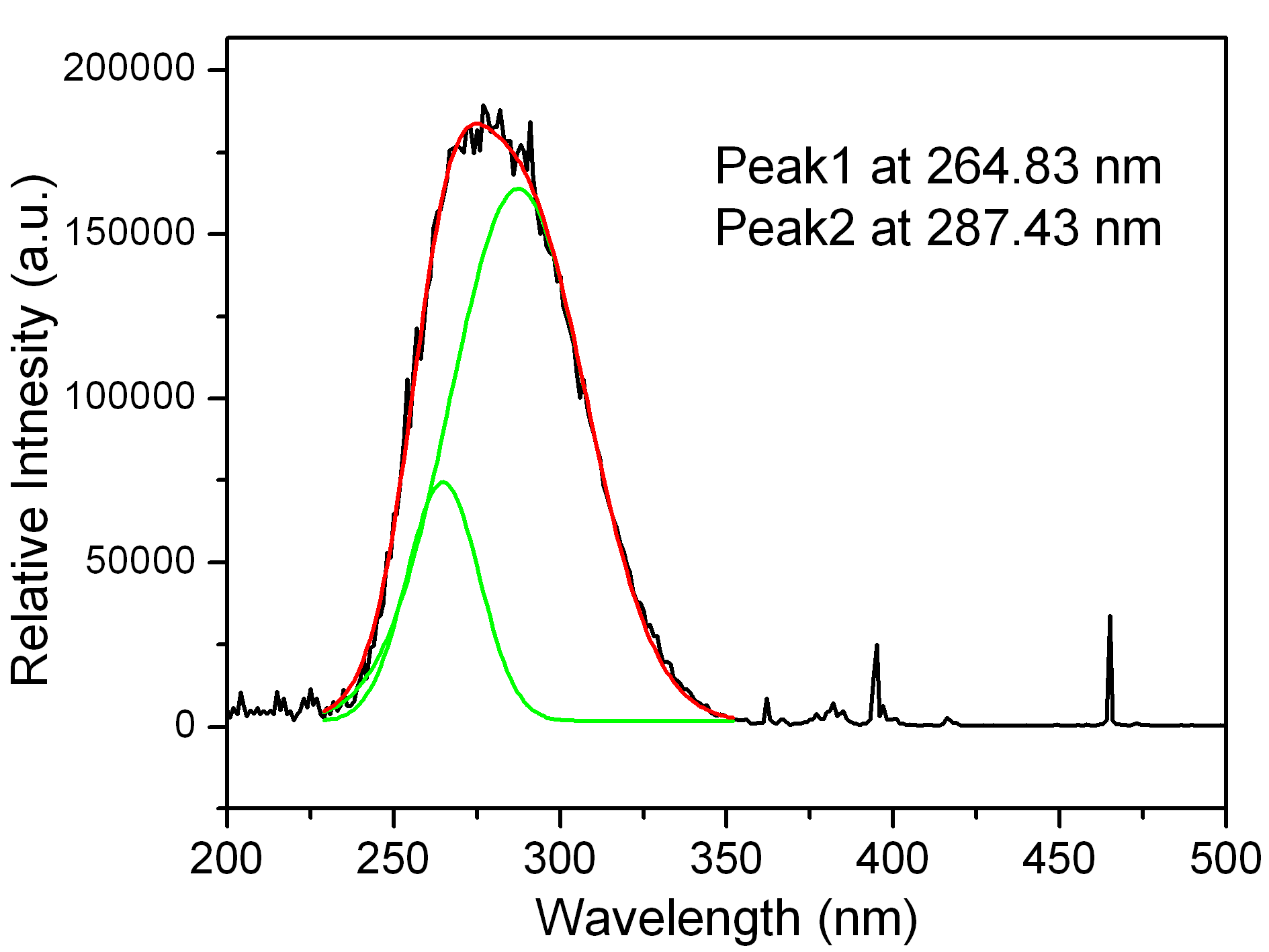


**Fig. S7** Fitting curves of the spectrum (i) in Fig. 5a by two Gaussian peaks.


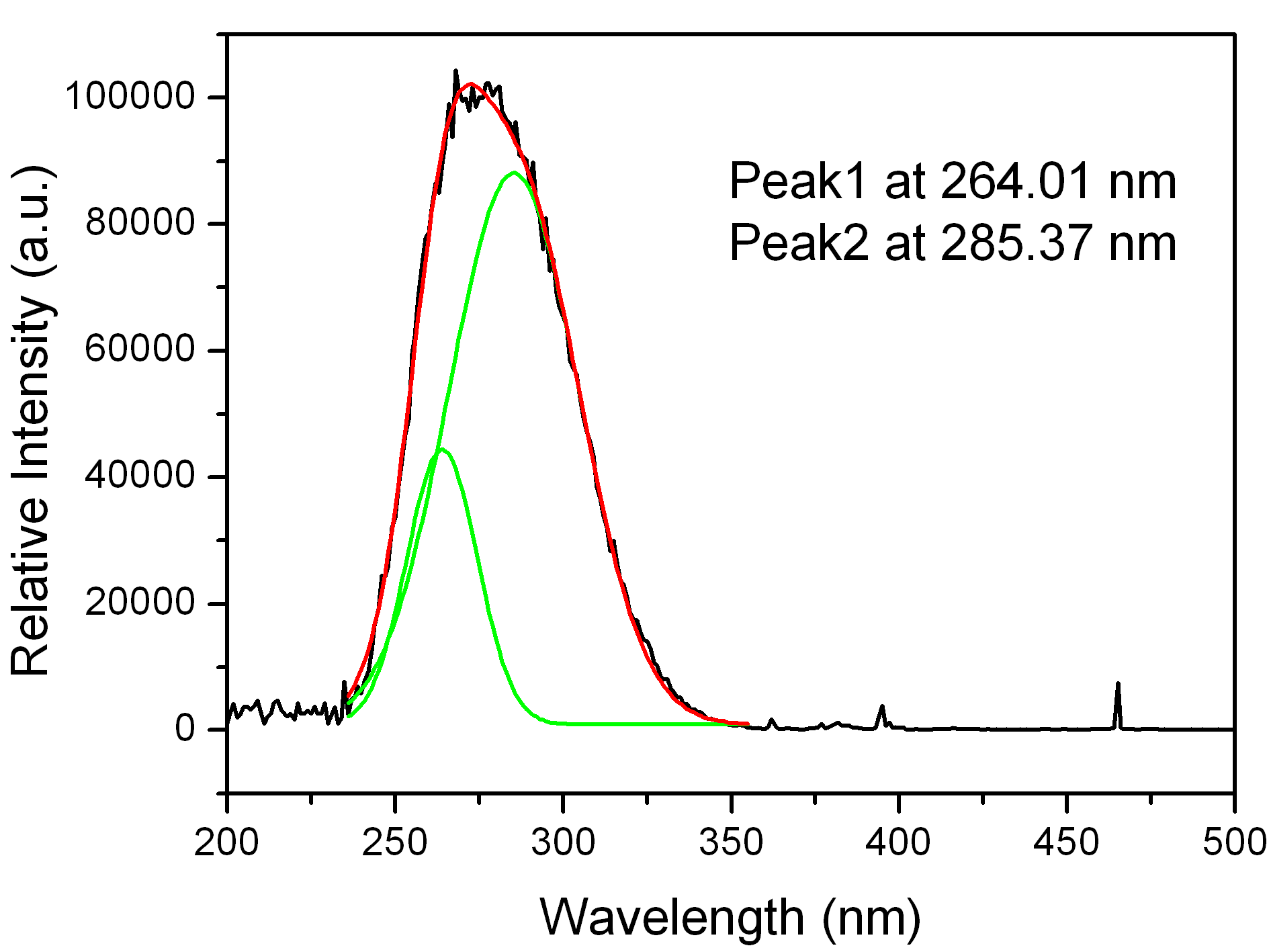


**Fig. S8** Fitting curves of the spectrum (vi) in Fig. 5a by two Gaussian peaks.


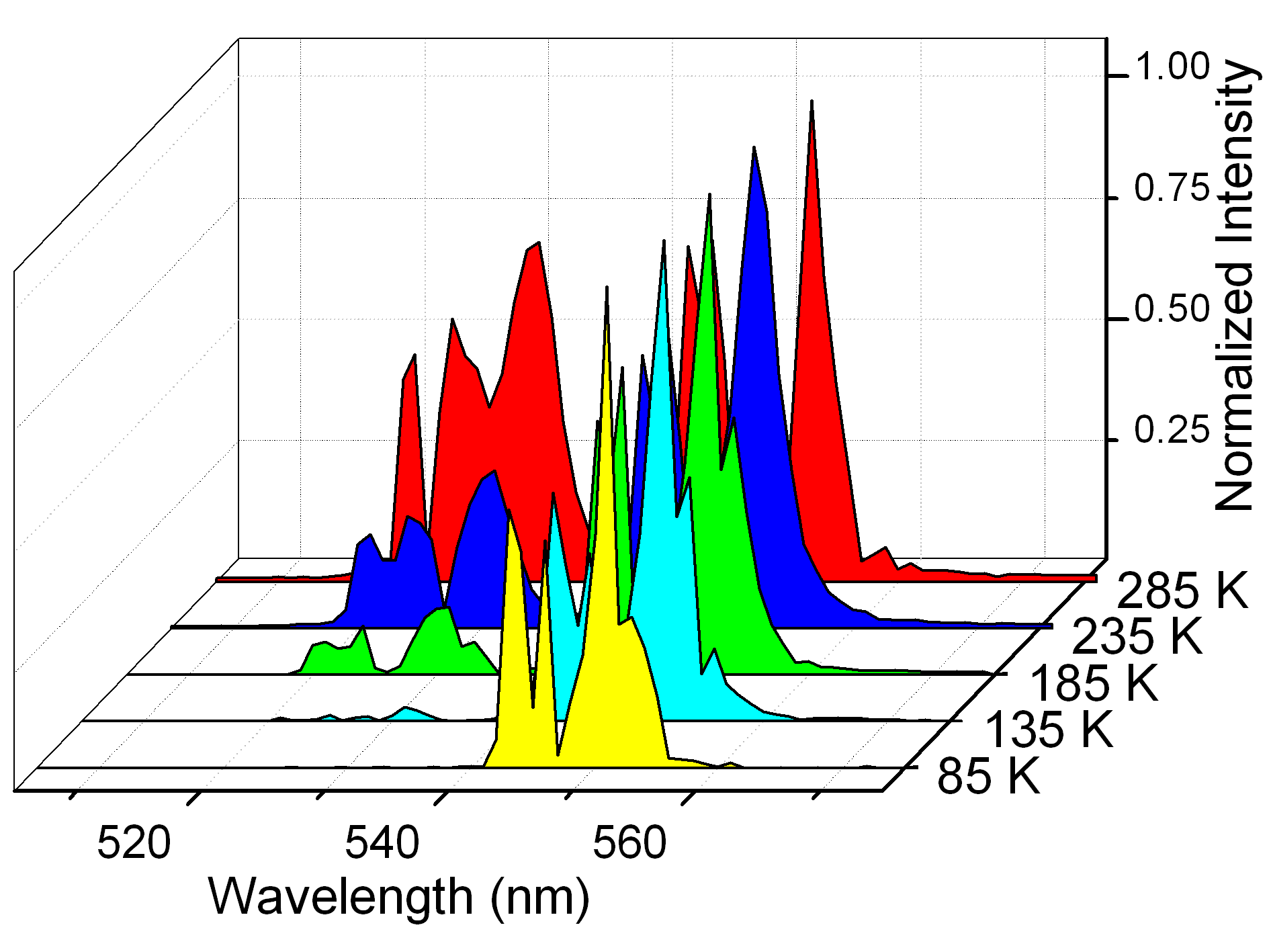


**Fig. S9** Temperature-dependent upconversion luminescence spectra (normalized to 1 at the maximum emission value) of NaGd(MoO_4_)_2_: 10%Yb^3+^/1%Er^3+^ square plate microcrystals from 85 to 285 K.


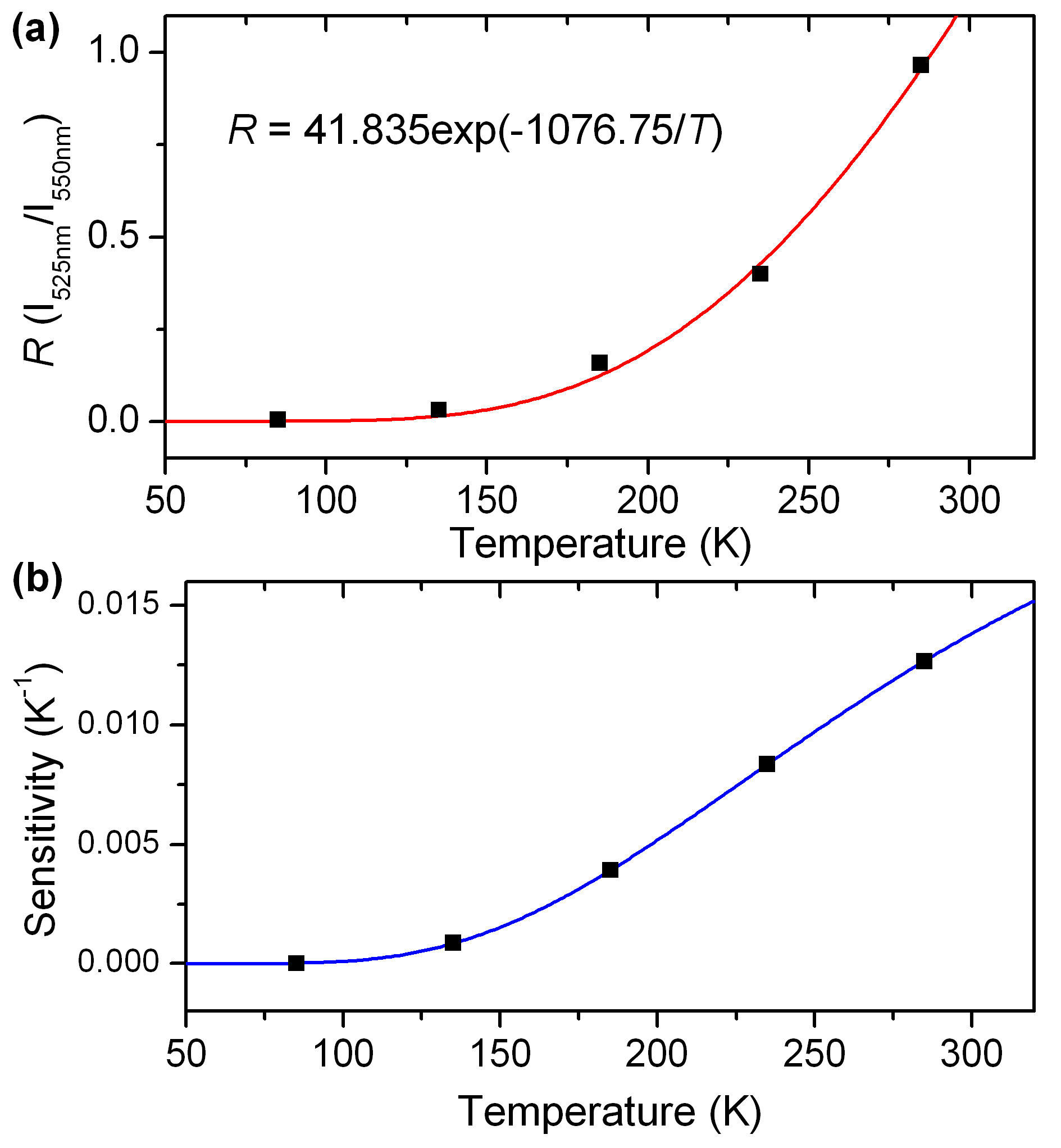


**Fig. S10** Dependence of intensity ratio *R* and thermometric sensitivity of NaGd(MoO_4_)_2_: 10%Yb^3+^/1%Er^3+^ square plate microcrystals on temperature. The sensitivity at 285 K is 0.01268 K^−1^.
